# Rethinking capacity building for knowledge mobilisation: developing multilevel capabilities in healthcare organisations

**DOI:** 10.1186/s13012-014-0166-0

**Published:** 2014-11-15

**Authors:** Roman Kislov, Heather Waterman, Gill Harvey, Ruth Boaden

**Affiliations:** Manchester Business School, The University of Manchester, Room D38 MBS East, Booth Street West, Manchester, M15 6PB UK; School of Nursing, Midwifery and Social Work, The University of Manchester, Jean McFarlane Building, Oxford Road, Manchester, M13 9PL UK; School of Nursing, The University of Adelaide, Level 3, Eleanor Harrald Building, Adelaide, 5005 SA Australia

**Keywords:** Capacity building, Capability development, Organisational capabilities, Organisational learning, Knowledge mobilisation, Knowledge translation, Knowledge transfer, Implementation, Service improvement, Facilitation

## Abstract

**Background:**

Knowledge mobilisation in healthcare organisations is often carried out through relatively short-term projects dependent on limited funding, which raises concerns about the long-term sustainability of implementation and improvement. It is becoming increasingly recognised that the translation of research evidence into practice has to be supported by developing the internal capacity of healthcare organisations to engage with and apply research. This process can be supported by external knowledge mobilisation initiatives represented, for instance, by professional associations, collaborative research partnerships and implementation networks. This conceptual paper uses empirical and theoretical literature on organisational learning and dynamic capabilities to enhance our understanding of intentional capacity building for knowledge mobilisation in healthcare organisations.

**Discussion:**

The discussion is structured around the following three themes: (1) defining and classifying capacity building for knowledge mobilisation; (2) mechanisms of capability development in organisational context; and (3) individual, group and organisational levels of capability development. Capacity building is presented as a practice-based process of developing multiple skills, or capabilities, belonging to different knowledge domains and levels of complexity. It requires an integration of acquisitive learning, through which healthcare organisations acquire knowledge and skills from knowledge mobilisation experts, and experience-based learning, through which healthcare organisations adapt, absorb and modify their knowledge and capabilities through repeated practice. Although the starting point for capability development may be individual-, team- or organisation-centred, facilitation of the transitions between individual, group and organisational levels of learning within healthcare organisations will be needed.

**Summary:**

Any initiative designed to build capacity for knowledge mobilisation should consider the subsequent trajectory of newly developed knowledge and skills within the recipient healthcare organisations. The analysis leads to four principles underpinning a practice-based approach to developing multilevel knowledge mobilisation capabilities: (1) moving from ‘building’ capacity from scratch towards ‘developing’ capacity of healthcare organisations; (2) moving from passive involvement in formal education and training towards active, continuous participation in knowledge mobilisation practices; (3) moving from lower-order, project-specific capabilities towards higher-order, generic capabilities allowing healthcare organisations to adapt to change, absorb new knowledge and innovate; and (4) moving from single-level to multilevel capability development involving transitions between individual, group and organisational learning.

## Introduction

Knowledge mobilisation (KM) is an emerging field of inquiry that seeks to strengthen connections between research, policy and practice across sectors, disciplines and countries, attempting to harness the benefits of research for organisational and societal improvement [1]. Challenges of KM between communities of researchers and practitioners have been of particular concern within healthcare research and policy, with a number of terms, such as knowledge utilisation, knowledge transfer, knowledge translation and knowledge exchange, used to describe the process of bridging the ‘gap’ between research and practice [2],[3]^a^. KM activities often take the form of relatively short-term projects dependent on limited funding, which raises issues about the long-term sustainability of KM and quality improvement designed, facilitated and supported by these initiatives within healthcare organisations [4],[5]. In the light of this, it is becoming increasingly recognised that the translation of research evidence into practice undertaken by KM initiatives has to be supported by developing the internal capacity of healthcare organisations to engage with and apply research, with ‘capacity building’ seen by many as one of the key aims of KM [6]-[10].

At the same time, most of existing health services research literature on capacity building tends to focus on developing skills needed to *undertake* research projects, with the development of skills required to successfully *utilise* research in clinical practice and quality improvement receiving relatively less attention (see [11]-[16] as examples of previous empirical and conceptual work in this area). Often, this literature does not explicitly link with broader social science and management theories exploring the complex, context-dependent and power-laden nature of learning within and between professional and organisational groups (see [17]-[20] for the importance of theory in KM research and practice). Drawing on empirical and theoretical literature on organisational capabilities and organisational learning, this paper advocates a practice-based, multilevel approach to capacity building through developing relevant capabilities at different levels of complexity. It proposes a number of theory-informed principles that could be useful for guiding *intentional* capacity building in healthcare organisations which is orchestrated by various KM initiatives undertaken by health professional associations [13], research foundations [21],[22], knowledge brokering programmes and networks [23]-[25] and collaborative partnerships bringing together healthcare professionals, researchers and KM experts [26]-[31]^b^.

The discussion is structured in the following way. The first section clarifies the definition of capacity building for KM, conceptualises capacity building as the process of developing skills, or capabilities, and proposes a typology of capabilities for KM. The second section uses the literature on dynamic capabilities to discuss mechanisms of capacity building at organisational level of analysis and various contextual factors influencing this process. The third section draws on the literature on intra-organisational learning to reflect on the interplay between individual, group and organisational levels of capability development within healthcare organisations. Four principles of the practice-based, multilevel approach to capacity development for KM advocated by the paper are formulated in the fourth section. The concluding section reiterates the key messages of the paper, presents the implications for evaluating capacity building programmes, discusses the limitations of the authors’ approach and suggests potential avenues for future research.

## Discussion

### Defining and classifying capacity building for KM

The notion of *capacity building* entered the academic international aid and development discourse in the 1970s [32] and has since spread to other domains, including public sector management and related disciplines [33]. Initially associated with achieving macro-economic growth in the developing world through ‘institution building’ and technological transfer, capacity building activities are now increasingly applied to individuals, organisations and communities and broadly refer to the creation, expansion or upgrading of a stock of desired qualities and features called *capabilities* that could be continuously drawn upon over time [34]. Health services research literature predominantly discusses *research capacity building* which is defined as ‘a process of individual and institutional development which leads to higher levels of skills and greater ability to perform useful research’ [35], with most commonly accepted outcomes including publications, conference presentations, successful grant applications and qualifications obtained [36]. Research capacity building, due to an acknowledged gap between producers and users of research [37], may not necessarily directly translate into improved outcomes for health services and patient care unless supported by an appropriate context-specific strategy for KM [38]. However, in focusing on research production, the notion of research capacity building does not explicitly take into account the importance of developing capabilities specific for research implementation, service improvement and KM.

This paper specifically focuses on *capacity building for KM* in healthcare organisations*,* which includes implementation of research evidence in practice. Drawing on the concept analysis of capacity building undertaken by Condell and Begley [39], we propose the following definition:

Capacity building for KM is a dynamic activity that augments capabilities to carry out functions or achieve objectives of KM programmes over the long term, leading to an improved provision of evidence-based healthcare.

A number of authors [32],[40] have shown that the concept of capacity building overlaps with the notion of sustainable change, which refers to the continual presence in an organisation of all or most of the practices/activities of an intervention or programme [41]. While the comparison of these two theoretical concepts lies beyond the scope of this paper, it needs to be highlighted that capacity building focuses on developing individual and organisational capabilities, emphasises the dynamic nature of the process and underscores an ability to apply acquired capabilities to new clinical and improvement issues, challenges and targets [32].

Previous research has shown that skills in collecting, appraising and disseminating research evidence are not enough for transforming research knowledge into clinical action [42],[43] and need to be accompanied by an understanding of professional behaviour [44],[45], local context [46],[47] and facilitation techniques [48],[49]. Practical implementation of healthcare KM initiatives, therefore, requires a wide range of capabilities that can be categorised into several overlapping knowledge domains, such as personal and organisational development, change management and diffusion of innovation [11] (Table 1).

**Table 1:** **Classification of KM capabilities according to knowledge domains**

Knowledge domain	Examples
1. Evidence management skills	• searching, appraising, storing and retrieving research evidence
	• synthesising research evidence
2. Process and system thinking	• ability to apply improvement methodology to address an issue
	• ability to ‘diagnose’ the broader context
3. Personal and organisational development	• theory and practice of group facilitation
	• stakeholder management and influencing skills
4. Involving patients, users, carers, staff and public	• service redesign based on patient and staff experience
	• identifying and acting upon stakeholders’ views and needs
5. Change management	• project and programme management skills
	• evaluating impact and learning
6. Delivering on cost and quality	• financial projection and calculation
	• measuring cost-effectiveness
7. Problem solving/consultancy	• problem identification, definition and structuring
	• written and visual presentation of data and recommendations
8. Diffusion of innovation	• assessing and evaluating potential innovations
	• building innovation into service improvement approaches

Source: Adapted and expanded from [11].

Organisational capacity to utilise research includes not only locating, obtaining and assessing the quality and relevance of research, but also ‘*organizational processes related to internalization, transformation and adaptation of research findings*’ [13]. To unpack the notion of organisational capacity for KM, the typology presented in Table 1 can be usefully complemented by an alternative categorisation of organisational capabilities according to their level of complexity [50] (Table 2), which is informed by the theoretical approach known as the resource-based view of the firm [51]-[53]. In the context of KM, *first-order (ordinary)* capabilities can be seen as an ability to deploy resources (such as time, technology or funds) to achieve relatively simple KM tasks. The emphasis of *second-order (core)* capabilities is on the integration of resources and ordinary capabilities into more complex KM projects aligned with an organisation’s strategic priorities. Finally, *third-order (dynamic) capabilities* denote an organisation’s ability to generate, extend and modify its lower-order capabilities to improve effectiveness and respond to the changing environment [54]-[56]. These may include, for instance, adapting existing KM projects to the changing context; absorbing, assimilating and exploiting KM skills acquired from external organisations and projects; and reconfiguring available skills and resources in order to generate new, innovative KM activities.

**Table 2:** **Classification of capabilities according to the level of complexity**[50]

Level	Type of capabilities	Definition	Examples related to KM
Zero-order	Resources	Stocks of available factors that are owned or controlled by the organisation [57]	Access to evidence
			Protected time of the clinical staff to get involved in service improvement
			Funds provided by external KM programmes
First-order	Ordinary capabilities	Abilities to deploy resources to fulfil relatively simple tasks	Using a case-finding tool to identify all patients with a certain chronic condition in the general practice system
Second-order	Core capabilities	Bundles of an organisation’s resources and first-order capabilities which are strategically important to achieving its objectives at a certain point in time	Undertaking audit and feedback of chronic disease registers in order to improve evidence-based management of patients and increase financial gains of the general practice
Third-order	Dynamic capabilities	Abilities to constantly integrate, reconfigure, renew and reconstruct an organisation’s resources and core capabilities in response to the changing environment	Ability to change the way audit and feedback is conducted in response to the changing research evidence and/or performance targets
			Ability to incorporate new research evidence, health improvement methodologies and other forms of knowledge to modify existing and design new KM projects
			Ability to design a new register verification tool enabling a quicker and hence more cost-effective way of conducting audit and feedback

The interplay between different levels of KM capabilities can be illustrated by the following example from our previous research [58],[59]:

Primary-care-based multiprofessional teams, comprising a general practitioner, nurse and practice manager, worked on a project aiming to improve identification and management of patients with chronic kidney disease (CKD). Supported by external knowledge mobilisation experts, members of these teams not only developed lower-order, project-related capabilities (such as verifying the CKD practice registers using the latest guidelines), but were also able to integrate them into a more generic second-order capability of conducting audit informed by improvement methodologies [27]. As a result of developing third-order, dynamic capabilities (such as the ability to transfer learning across teams and the ability to adapt to external change), improvements in some general practices occurred even in areas not related to the initial focus on CKD. These positively affected other disease registers, overall communication within the practice and procedures for the updating of treatment protocols.

The next section will use insights from literature on dynamic capabilities to explore the mechanisms underpinning the development of KM capabilities in healthcare organisations and discuss contextual factors that can influence this process.

### Mechanisms of capability development in organisational context

The following subthemes can be identified from the literature.

#### Repeated use of capabilities in practice

Both lower-order and higher-order capabilities strengthen with repeated use [60]. Higher-order capabilities are affected by and operate on lower-order capabilities: the former are often combinations of simpler, foundational capabilities that must be learned first [53],[60]. Repeated practice (which can be codified in the form of technology and formal procedures) and learning from past mistakes are seen as important mechanisms underpinning the evolution of capabilities [53],[61]. It should however be noted that the repeated use of lower-order capabilities without change, i.e. without developing higher-order, dynamic capabilities, may render lower-order capabilities more difficult to change in the future, with an organisation becoming trapped in existing ways of doing things [60],[61]. For instance, drawing on the examples presented in Table 2, it could be argued that an ability to perform casefinding on a practice register for a certain condition (an ordinary capability) will only lead to the identification of appropriate cases in the long run if accompanied by an ability to regularly update the case-funding algorithm in line with changing research evidence and diagnostic criteria (a dynamic capability). On the other hand, developing and using dynamic capabilities is costly in the short term as it involves the consumption of organisational resources in integrating, reconfiguring and altering existing lower-order capabilities [60].

#### Combining acquisitive and experience-based learning

It has been suggested that the development of capabilities is governed by two distinct but complementary learning processes [61],[62]:

- *Acquisitive learning*, which occurs when an organisation acquires and internalises knowledge from other organisations, new employees and external consultants or by systematically scanning the environment for relevant information;
- *Experience-based learning*, which happens internally and generates new knowledge that is distinctive to the organisation; it can happen through improvisation, trial and error and experimentation.

Although acquisitive learning plays an important role during early stages of capability formation by providing innovative insights and new opinions, what is learned from others needs to be connected with existing organisational capabilities, structures and processes. Refining, developing and adapting capabilities to the specific organisational contexts are best achieved through experience-based learning, i.e. learning-by-doing, its quality being dependent on the amount of effort spent on analysing the experience obtained from past cases and capturing the lessons learnt [61].

#### An interplay between tacit and explicit knowledge

Developing organisational capabilities involves a two-way relationship between explicit and tacit knowledge [63]. Explicit, articulated, codified knowledge (sourced, for instance, from outside an organisation) needs to be fed into organisational members, applied in practice and made tacit. At the same time, organisational capabilities can also be preserved in the less explicit forms, such as history, norms, beliefs and values that are conveyed to a succession of organisational members through early socialisation and longer-term accumulated social interactions [64],[65]. To become more readily available to organisational members, the tacit, unspoken, taken-for-granted components of experience-based knowledge often need to be formalised through the processes of articulation and codification [54]. *Knowledge articulation* refers to the process by which individuals make their tacit experiential knowledge explicit by expressing their opinions and beliefs, engaging in constructive confrontations and challenging each other’s viewpoints. *Knowledge codification* is a step beyond knowledge articulation and involves individuals codifying their understanding of internal organisational processes in the form of manuals, protocols, decision support systems and other written and electronic tools. It is important to remember, however, that an over-reliance on articulated and codified knowledge can limit responsiveness to the ever-changing context and impair the realisation of dynamic capabilities in practice if used without reflection [61]. Keil [61] suggests that articulation and codification as learning mechanisms should be complemented by *knowledge exchange* through social networks that can be intra-organisational or cut across organisational boundaries and can be based both on informal relationships and formal events.

#### Moderating influence of organisational context

Management literature on capability development and health literature on research capacity building identify a number of contextual factors that influence the process of learning new skills in organisations. Some of these factors refer to organisational features, such as organisational culture and attitudes to change and learning [54],[66],[67]; senior management support of learning and capability development [12],[68],[69]; the presence of a coherent change strategy [47],[70]; distributed leadership with credible opinion leaders; and good relationships between managers and clinicians [47],[71]. Existing knowledge and capacity in a given area are seen as an advantage for further capacity building [66], with many organisations which successfully participate in quality improvement initiatives demonstrating a strong preexisting orientation towards innovation, improvement and change [72]. Participation in effective networks, partnerships and collaborations can also influence organisational learning and capacity building [36],[40],[69]. Finally, developing capabilities requires investing resources, for instance allocating protected time for staff to engage in activities leading to capacity building [68] or putting in place appropriate award systems [69].

To conclude, capability development in organisations happens through repeated practice and involves an integration of acquisitive learning, i.e. getting ideas from the outside, and experience-based learning, which happens internally. An integration of acquisitive and experience-based learning within organisations is aided by the mechanisms of knowledge articulation, codification and exchange, whereby newly developed capabilities get refined, modified and spread across the whole organisation. The process of capability development within organisations is also highly contingent on the organisational features, resource endowments and starting learning positions, all of which vary widely across organisations. As can also be inferred from this section, literature on dynamic capabilities deploys *organisation* as the main unit of analysis. Less emphasis has been put here on the *intra-organisational* processes of intentional capacity building induced by external KM initiatives with which healthcare organisations collaborate. The next section will try to address this gap by exploring the literature on intra-organisational learning, specifically focusing on the interplay between individual, group and organisational levels of capability development.

### Individual, group and organisational levels of capability development

Intentional capacity building undertaken by KM initiatives in healthcare organisations needs to be analysed as *integration* of existing organisational capabilities and the developmental input offered by external KM experts. This integration implies a combination of ongoing experience-based learning taking place within a healthcare organisation and acquisitive learning through which an organisation acquires new resources and capabilities from a KM initiative. This process is not conflict-free and often requires formal (e.g. strategic planning and resource allocation procedures) and informal (e.g. negotiating and mediating) efforts to resolve disagreements about the nature and scope of capabilities to be developed, resources to be allocated and priorities to be selected [60],[73]. In addition to broader contextual factors described in the previous section, the process of integration is affected by the fact that quality improvement, health promotion and other KM activities often take the form of separate, time-limited projects with clearly defined targets and outcomes, rather than ongoing long-term or integrated programmes of work [4],[74]. This raises a question about the extent of organisational learning from standalone projects [75]-[77].

Building on previous literature on capacity building in health services research [36],[78] and our own experience [58],[59],[79],[80], we suggest that capability development at the interface between a healthcare organisation and a KM initiative can unfold in three ‘configurations’: *individual-centred, team-centred and organisation-centred* (Table 3). These differ by the ‘entry point’ that the KM initiative uses to tap into the internal processes within a healthcare organisation.

**Table 3:** **Approaches to capability development in healthcare organisations undertaken by external KM initiatives**

Starting point	Description	Example
1. Individual-centred	An individual based in a healthcare organisation is supported by or embedded into an external KM team	Training and supporting secondary care based heart failure nurses to undertake audit and feedback of heart failure care facilitated by a KM team in general practices [80]
2. Team-centred	A team based in a healthcare organisation is working on a KM project supported by an external KM initiative	Training and supporting a multiprofessional team to undertake an evidence-based improvement project around identification and management of patients with CKD [58],[59]
3. Organisation-centred	The whole organisation is involved in one or several KM projects supported by an external KM team	Supporting all staff members of a general practice (i.e. not just a nominated ‘lead’ or ‘improvement team’) to actively participate in service improvement projects facilitated by KM experts

Although the literature on capacity building in healthcare advocates a ‘whole of organisation’ approach that integrates all levels of capacity building [69],[81], this tends to be more resource-intensive than individual- and team-centred approaches. Arguably, it is more likely to succeed in relatively small organisations with a long-term strategic orientation to learning and improvement, established intra-organisational channels of communication and absence of strong intra-organisational barriers [59],[67]. On the other hand, using individual- and team-centred approaches to capacity building raises questions about the subsequent assimilation of acquired knowledge and capabilities (i.e. individual and team learning) into broader organisational learning, which is a nested phenomenon occurring at several different but interrelated levels at the same time, whereby learning at one level may substitute for, trade off against or even inhibit learning at another [76],[82]^c^.

Capabilities may be acquired individually but the knowledge of what constitutes ‘acceptable’ practice is developed and negotiated collectively within a group or community [65],[83]-[85]. At the same time, knowledge that is uniquely possessed by a member is less likely to be mentioned, repeated and attended to in group discussions than commonly held ‘collective’ knowledge [86]. As a result, organisations do not always ‘know what they know’: organisational members with information needs are frequently unaware of the existence of knowledge held by other members [87] and individual-centred capability development may become ‘stockpiled’ if the organisation has a limited capacity to absorb the learning [88]. Similarly, integration of team-specific knowledge and skills into the learning of the whole organisation may be hampered by divisional, departmental, and other intra-organisational barriers to knowledge sharing [59],[89]-[91]. Learning *within* project teams may not translate into learning *between* project teams that can enhance organisational learning [75]-[77].

Moving from individual to group to organisational learning involves continuing conversations among organisational members, participation in shared practices, institutionalisation of capabilities in the systems, structures, strategy and routines and investments in information systems and infrastructure [86],[88]. When facilitating the transitions between different levels of capability development, two tensions may emerge. First, learning by doing often tends to be favoured over learning from others, especially if the ‘others’ are unknown [92], which underscores the importance of relationships between the facilitators coming from external KM initiatives (especially where they are not known to the organisation previously) and practitioners working within healthcare organisations. In addition, organisational learning involves a tension between the embedded institutionalised learning from the past and the newly acquired learning [88],[93]. Capability development within organisations with a high degree of institutionalised learning can thus require the *unlearning* of previously established ways of doing things which have become irrelevant and counterproductive [16],[94]. At the individual and group levels, this could be facilitated through training, mentoring or reflection while organisational unlearning may involve restructuring and implementing new performance management, communication or resource allocation systems [95].

### Rethinking capacity building for KM: lessons for KM initiatives

In light of the analysis presented in the previous three sections, we suggest the following four principles underpinning a practice-based approach to developing multilevel KM capabilities in healthcare.

#### From ‘building’ towards ‘developing’ capacity

We suggest that ‘developing’ capacity may be a better term than ‘building’ capacity for the following reasons. Whilst the ‘building’ metaphor puts an emphasis on the creation of new capabilities undertaken by KM initiatives for the participating healthcare organisations, the notion of ‘capability development’ underscores the expansion and upgrade of the capabilities already existing in healthcare organisations. It could be argued that embarking on an externally supported KM programme already signifies an interest in procuring external knowledge to improve everyday practice, which can be interpreted as an innovative capability [72]. Similarly, through their previous experience, healthcare organisations already possess a wide array of ordinary and core capabilities that may be renewed, reconfigured and integrated in the process of KM. In addition, even though an external KM project can introduce some completely new skills to a healthcare organisation, they still need to be assimilated into the existing organisational routines, which includes modification and adaptation of externally acquired knowledge to local contexts. This process is affected by multiple factors, some of which (e.g. resources and facilitation) are more amenable to control by an external KM initiative than others (e.g. organisational culture and leadership) (see also [40]). The role of a KM initiative is, therefore, to tap into existing organisational capabilities and attempt to develop them by offering relevant skills, tools and resources, rather than build new skills in healthcare staff that may remain unused and lost after the project is completed.

#### From passive recipients towards active participants

Although formal training in quality improvement and KM can be an important component of capacity development, it will only be effective if directly connected with everyday practice [11],[74], with learning by doing or, in this context, ‘learning by practising KM’, acting as the main mechanism of learning taking place at the interface of a healthcare organisation and a KM initiative. In order for KM capabilities to be developed in healthcare organisations, healthcare professionals should be actively involved in KM projects as implementers of change, rather than act as passive recipients of change introduced by external KM experts. The role of the latter in the process of facilitating capacity development in healthcare organisations is the following:

- Providing resources (e.g. buying out clinicians’ time);
- Creating opportunities for healthcare staff to improvise, experiment and learn from mistakes whilst engaging in the practice of KM;
- Creating opportunities for healthcare staff to come together to exchange knowledge between individuals, groups and organisations;
- Providing continuous mentorship and support to healthcare staff;
- Providing specialist expertise when needed (e.g. complex data analysis techniques);
- Introducing theoretical and methodological tools, frameworks and ideas that can help healthcare staff to reflect on their practice and achieve an integration of old and new knowledge and skills (this is discussed in more detail in the following two subsections).

#### From lower-order towards higher-order capabilities

When KM projects tend to focus on a certain topic and have well-defined targets and outcomes, there is a risk that capabilities developed through participation in the project will remain project-specific and will not be transferred to other areas. In addition to project-specific, lower-order capabilities, emphasis should be put on the development of more generic, dynamic capabilities, i.e. an ability to change lower-order capabilities and thus better adapt to change, absorb external knowledge and innovate. As argued in the management literature [50],[52],[54],[56],[60], dynamic capabilities are especially important for organisations located in rapidly changing environments, of which a healthcare system could be an example [96]. KM initiatives can therefore enhance their effectiveness in a number of ways:

- Introducing healthcare staff to quality improvement methodologies and theories of change and innovation which, if continuously applied in practice, may enable the process of knowledge articulation seen as a prerequisite for the formation of dynamic capabilities;
- Supporting the process of knowledge codification, whereby generic lessons learnt throughout the KM project are summarised in the form of organisational manuals, toolkits and protocols, and helping healthcare organisations integrate these into their day-to-day routines in a reflective and context-sensitive way;
- Helping healthcare staff to reflect on the contextual features of their organisation, to use organisational channels of communication (such as meetings, newsletters or informal chats) for the sharing of KM-related knowledge and to utilise the relevant capabilities in other areas of work throughout the organisation.

#### From single-level towards multilevel learning

To maximise the effectiveness of intentional capacity building in healthcare organisations, KM initiatives must take into account the mechanisms that allow transitioning between individual, group and organisational learning. Regardless of the point of contact between a KM initiative and a healthcare organisation, it is important to pay attention to different levels of learning within the healthcare organisation. The following transitions may require support or facilitation:

- *Individual to group level of capability development*—enabling the spread of learning from individuals to groups;
- *Group to organisational level of capability development*—enabling the spread of learning between different groups and from groups to the broader organisation;
- *Organisational to individual level of capability development*—enabling the spread of institutionalised organisational learning to new organisational members^d^ (Table 4).

**Table 4:** **Facilitating the transitions between different levels of learning within a healthcare organisation**

Area	Actions to be considered by the facilitators
Transition from the individual to group level of learning	• Involving multiprofessional teams in KM projects
	• Encouraging the discussions of KM projects at formal and informal team meetings and other events
	• Using individual skills and knowledge to develop wider KM activities involving more staff
	• Enabling individual organisational members to act as educators for the rest of the organisational staff
Transition from the group to organisational level of learning	• Rotating organisational members between different teams and departments
	• Identifying and engaging individuals acting as intermediaries between different teams/departments
	• Helping KM teams present their work to the wider organisation
	• Institutionalising knowledge and skills in the form of organisational protocols, procedures and reminders
Transition from the organisational to individual level of learning	• Recruiting more staff from across an organisation to take part in KM activities
	• Creating opportunities for new staff to shadow more experienced organisational members
	• Raising awareness about the location of relevant KM skills within an organisation
	• Updating protocols and procedures in the light of the new knowledge and skills acquired by an organisation

In addition to supporting the interpretation, integration and institutionalisation of KM knowledge and skills, the following steps could be taken by KM facilitators to support multilevel capability development in healthcare organisations:

- Information about ongoing KM work (what, how and by whom is being done) should be widely distributed across a healthcare organisation because information distribution leads to more broadly based organisational learning [87];
- When planning for knowledge sharing within an organisation, emphasis needs to be placed on encouraging individuals and teams involved in KM projects to articulate *how* they have achieved their goals, not only *what* they have achieved, i.e. on procedural knowledge rather than product knowledge [92];
- When spreading KM skills and knowledge across an organisation, intermediaries could be involved: those who connect the external KM facilitators with different professional groups within an organisation [97], formally oversee several different teams [98] or fulfil an informal knowledge brokering role between different intra-organisational groups [99],[100].

## Conclusion

The four principles outlined above can be useful for a range of KM initiatives, such as Collaborations for Leadership in Applied Health Research and Care (CLAHRCs) [101] and Academic Health Science Networks (AHSNs) [102] in England; the Knowledge Network in Scotland [25]; the Accreditation Collaborative for the Conduct of Research, Evaluation and Designated Investigations through Teamwork (ACCREDIT) [103] in Australia; and Knowledge Translation Canada [104]. Table 5 presents the key questions to be addressed when implementing these principles in practice.

**Table 5:** **Questions to be addressed when applying the principles of KM capability development in practice**

Principle	Questions to be addressed
1. Moving from ‘building’ capacity from scratch towards ‘developing’ capacity of healthcare organisations	• What existing knowledge and skills within a healthcare organisation could be utilised for KM projects?
	• Where in the organisations are these knowledge and skills located?
	• How can these knowledge and skills be further developed?
	• What KM skills are currently lacking and how can their development be supported?
	• How will the newly acquired knowledge and skills integrate with existing ways of doing things within an organisation?
2. Moving from passive involvement in formal education and training towards active, continuous participation in KM practices	• What KM activities are the staff actively involved in?
	• How are the roles distributed between the external facilitators and the local staff involved in KM projects?
	• What arrangements are in place to enable the facilitative role of external KM experts?
	• What incentives can be provided to support the engagement of local staff in KM activities?
	• What mentorship and shadowing options are available for healthcare staff?
3. Moving from lower-order, project-specific capabilities towards higher-order, generic capabilities	• What mechanisms will ensure maintenance and further development of capabilities within an organisation?
	• How will project-specific knowledge and skills be transferred to other areas of practice?
	• What theoretical models, frameworks and approaches can be useful to guide the local development of KM capabilities?
	• What are the arrangements for updating organisational protocols, guidelines and procedures related to KM?
	• What are the arrangements for identifying new learning opportunities outside an organisation?
4. Moving from single-level towards multilevel learning about KM within healthcare organisations	• How do the capabilities developed by individual and teams link with organisational priorities?
	• What are the intra-organisational boundaries to sharing knowledge and skills and how are these boundaries going to be addressed?
	• How is sharing knowledge and skills within the project team and between the teams going to be supported?
	• What mechanisms are in place to ensure the unlearning of irrelevant knowledge?
	• What arrangements are in place to ensure that the whereabouts of relevant knowledge and skills in an organisation are known to its members?

This paper calls for a shift from a focus on the content of capacity building programmes (i.e. what topics should be covered and what capabilities have to be developed) towards a more strategic thinking about the subsequent ‘life’ of these capabilities within a recipient healthcare organisation (i.e. how these knowledge and skills will be utilised, maintained and updated as part of organisational ways of doing things). We have emphasised the importance of integrating ‘learning from others’, through which healthcare organisations acquire knowledge and skills from KM experts, and ‘learning by doing’, through which healthcare organisations adapt, absorb and modify their knowledge and capabilities through repeated practice. We have also proposed that capacity building activities should go beyond supplying a range of training opportunities to healthcare staff and incorporate an active facilitation of the processes by which newly acquired knowledge and capabilities are going to be integrated into the wider organisation, involving the transitions between individual, group and organisational learning.

A detailed exploration of criteria to be used when monitoring and assessing capacity development in healthcare organisations involved in KM programmes lies beyond the scope of this paper. However, our analysis has a number of implications for theory-informed evaluations of capacity development. The outcomes of developing KM capabilities, i.e. acquired capabilities and resulting organisational change, are difficult to quantify, while such measures as the number of KM projects undertaken by the staff or the number of individuals trained are unlikely to capture the ‘soft’ and complex nature of capacity development. A range of qualitative process descriptors should therefore be deployed, with their selection driven by the local context of KM, taking into account the following dimensions:

- Evidence of KM skills developed through practice;
- Ability to independently exercise, develop and modify these skills after the project is over;
- Applicability of the capabilities developed in a certain project to other projects or clinical areas;
- Uptake of capabilities developed by individuals and teams across the whole organisation;
- Impact of the capabilities on organisational strategy, culture and procedures.

Whilst developed from previous research in related areas, the analysis presented in this paper and our conclusions must be regarded as tentative until corroborated by rigorous empirical evidence. The paper limits the discussion of capacity building to capabilities, i.e. skills, whereas capacity building tools, infrastructure, systems and roles [105] have not been explicitly addressed. Its focus is on developing capabilities in healthcare organisations, while capacity development within KM initiatives supporting these organisations has not been discussed and can provide an interesting direction for future research. Other topics for empirical explorations may include: the comparative effectiveness of different strategies aiming to facilitate the transition between individual, group and organisational learning; the relationship between individual characteristics of healthcare and KM staff (e.g. their confidence, status, legitimacy or position in organisational and professional networks) and the trajectories of KM capability development; and the interplay between capability development in healthcare organisations and the type and scope of facilitation approach chosen by the external KM initiative.

## Endnotes

^a^Following Ferlie and colleagues [20], we prefer the term ‘KM’ to other related concepts (e.g. ‘knowledge management’, knowledge transfer’, ‘research utilisation’, etc.) because it is looser and signals possible resistance or unplanned outcomes (such as, for instance, the formation of cross-project barriers to learning within KM initiatives [89]).

^b^Intentional capacity building activities are specifically designed to develop relevant knowledge and skills, whereas unintentional capacity building refers to learning benefits accrued in a more random way by virtue of participation in a KM programme [106].

^c^Mechanisms enabling the transitions between individual, team and organisational levels of learning are not dissimilar to the fundamental mechanisms underpinning the formation of dynamic capabilities explored in the previous section’see, for instance, Newell and Edelman [98], who conceptualise cross-project learning as a dynamic capability.

^d^Moving from organisational to individual level of learning is mediated by the group level and involves the conversion of explicit knowledge into tacit through dialogue and shared practice [88] discussed earlier.
